# The Role of Cannabinoid Receptors in the Descending Modulation of Pain

**DOI:** 10.3390/ph3082661

**Published:** 2010-08-16

**Authors:** Enza Palazzo, Livio Luongo, Vito de Novellis, Francesco Rossi, Sabatino Maione

**Affiliations:** Department of Experimental Medicine, Pharmacological Division, the Second University of Naples, via Costantinopoli 16, 80138 Naples, Italy; E-Mails: livioluongo@inwind.it (L.L.); vito.denovellis@unina2.it (V.D.N.); francesco.rossi@unina2.it (F.R.); sabatino.maione@unina2.it (S.M.)

**Keywords:** periaqueductal grey, rostral ventromedial medulla, antinociceptive descending pathway

## Abstract

The endogenous antinociceptive descending pathway represents a circuitry of the supraspinal central nervous system whose task is to counteract pain. It includes the periaqueductal grey (PAG)-rostral ventromedial medulla (RVM)-dorsal horn (DH) axis, which is the best characterized pain modulation system through which pain is endogenously inhibited. Thus, an alternative rational strategy for silencing pain is the activation of this anatomical substrate. Evidence of the involvement of cannabinoid receptors (CB) in the supraspinal modulation of pain can be found in several studies in which intra-cerebral microinjections of cannabinoid ligands or positive modulators have proved to be analgesic in different pain models, whereas cannabinoid receptor antagonists or antisense nucleotides towards CB1 receptors have facilitated pain. Like opioids, cannabinoids produce centrally-mediated analgesia by activating a descending pathway which includes PAG and its projection to downstream RVM neurons, which in turn send inhibitory projections to the dorsal horn of the spinal cord. Indeed, several studies underline a supraspinal regulation of cannabinoids on γ-aminobutyric acid (GABA) and glutamate release which inhibit and enhance the antinociceptive descending pathway, respectively. Cannabinoid receptor activation expressed on presynaptic GABAergic terminals reduces the probability of neurotransmitter release thus dis-inhibiting the PAG-RVM-dorsal horn antinociceptive pathway. Cannabinoids seem to increase glutamate release (maybe as consequence of GABA decrease) and to require glutamate receptor activation to induce antinociception. The consequent outcome is behavioral analgesia, which is reproduced in several pain conditions, from acute to chronic pain models such as inflammatory and neuropathic pain. Taken together these findings would suggest that supraspinal cannabinoid receptors have broad applications, from pain control to closely related central nervous system diseases such as anxiety and depression.

## 1. Cannabinoid Receptors and Analgesia

Basic research indicates that endocannabinoids play a well-established role in alleviating behavioral responses to acute, inflammatory and neuropathic pain [1,2,3]. A large body of studies using receptor deletion and selective agonists and antagonists have suggested that analgesia is due to the activation of both cannabinoid subtype 1 and 2 (CB1 and CB2) receptors [4,5,6,7]. The role of CB1 receptor in modulating pain is confirmed by its wide distribution within the key sites of pain neuraxis. CB1 receptors are densely expressed on the superficial layers of spinal dorsal horn, the dorsal root ganglia, the peripheral terminals of primary afferent neurons and within the pain descending pathway [8]. CB2 receptors are mainly located in the immune system therefore representing a target in inflammatory pain processing. Although early studies did not find CB2 receptors in the central nervous system, recent works have reported the presence of CB2 mRNA within the spinal cord [9] and CB2 receptor protein within the brain [10,11]. Consequently CB2 expression within the brainstem, cortex and cerebellum has been confirmed at this point [11]. The functional role of CB2 receptors in the CNS is however still unclear since a functional imaging study has demonstrated that CB2 receptor antagonism did not modify brain activation evoked by systemic administration of a non-selective cannabinoid agonist [12] suggesting that CB2-mediated cannabinoid-induced changes in brain activity are not functionally active under normal physiological conditions.

The wide distribution of CB receptor within the main pain processing sites offers an unlimited potential for inducing analgesia in several pain conditions. Stimulation of spinal cord CB1 receptor has been shown to inhibit nociceptive responses in *in vivo* and *in vitro* studies [13,14,15,16,17,18]. The contribution of peripheral CB1 receptors in analgesia has been assessed in a recent study in which CB1 receptors were selectively ablated in primary nociceptive sensory neurons with no change in CB1 receptors elsewhere, including the central nervous system. Resulting mice were phenotypically hypersensitive to noxious heat or mechanical stimuli and developed increased neuropathic pain by nerve injury. The same mice were unresponsive to locally or systemically administered cannabinoids, while they instead responded to those intrathecally administered [19].

Apart from CB1 and CB2 receptors, anandamide and other “endocannabinoids” such as *N*-arachidonoyl dopamine (NADA) activate the transient receptor potential vanilloid type 1 (TRPV1) channel [20,21,22]. TRPV1 is a non selective cation channel belonging to the large family of transient receptor potential (TRP) channels which is activated by noxious heat (>42 °C), low pH (>6.0) and by the red hot chilli pepper constituent capsaicin. Anandamide has been recently shown to excite C-fibers and to produce nociceptive behaviour via TRPV1 activation [23]. Moreover, prostaglandins and bradykinins increase TRPV1 sensitivity to anandamide under inflammatory conditions [24]. Transient receptor potential vanilloid type 4 (TRPV4) channel is also activated by anandamide and arachidonic acid metabolites produced from anandamide hydrolysis and CYP450 oxygenases [25,26]. Its stimulation promotes the release of substance P and CGRP neuropeptides from central projections of primary afferents in the spinal cord [27]. Its role in detecting warm temperatures and in chemically induced thermal hyperalgesia has been confirmed by TRPV4^−/−^ mice which showed markedly reduced carrageenan and capsaicin-induced hyperalgesia [28]. Moreover, under certain conditions, such as inflammatory pain, functional interaction between transient receptor potential ankyrin type 1 (TRPA1) and TRPV1 channels leads to the integration of inflammation-induced stimuli by sensory neurons. Inflammation-induced activation of the TRPA1 channel is controlled by the TRPV1 channel and by diacylglycerol (DAG) and 2-AG, the effects of which are also greatly enhanced via functional interaction with TRPV1. Activation of TRPV1 and TRPA1 leads to desensitization and consequent behavioral analgesia. Cannabinoids and/or endocannabinoids gate, directly and indirectly, at least five TRP channels, although they seldom act as full agonists [29,30] so that the TRP-mediated responses may not reach the threshold level to induce nociceptor excitation. By this subject these TRP channels are often referred as ionotropic cannabinoid receptors (ICR). 

## 2. Cannabinoid Receptors and Supraspinal Pain Modulation

Supraspinal sites of cannabinoid-induced antinociception were initially revealed by indirect studies in which the antinociceptive effects of Δ^9^-THC [16] or the effects of systemically administered cannabinoids on noxious stimulus*-*evoked responses [31] were preserved following spinal transections. Direct evidence of supraspinal sites of cannabinoid analgesic action has been provided by the observation that intraventricular administration of cannabinoid agonists, such as WIN55212-2, CP55940 and Δ^9^*-*THC, induced antinociception [32,33]. The identification of supraspinal sites of cannabinoid-induced antinociception has been made possible by site-specific injections of cannabinoid agonists to various brainstem areas. Microinjections of cannabinoids into sites such as the dorsolateral periaqueductal grey (PAG), dorsal raphe nucleus (DRN), rostral ventromedial medulla (RVM), amygdala, lateral posterior and submedius regions of the thalamus, superior colliculus, and noradrenergic A5 region have been shown to produce antinociceptive effects [34,35,36,37]. 

## 3. Cannabinoid Receptors in Pain Descending Pathway

The descending nociceptive modulatory system represents an endogenous antinociceptive system, whose electrical or chemical activation counteracts pain. Its discovery dates back to 1969 when Reynolds [38] observed that PAG electrical stimulation produces strong analgesia in rats. It is now well established that this brainstem*-*spinal pathway inhibits pain (for comprehensive reviews see 39-41). A potential treatment for silencing pain is therefore the activation of the PAG-RVM-dorsal horn (DH) circuitry, which is the best characterized pain modulation system through which pain is endogenously inhibited. PAG and RVM are major sites for opioid-induced analgesia. Apart from opioid receptors, the PAG and RVM also contain cannabinoid CB1 receptors [28,29,30]. Functional studies have shown that the microinjection of cannabinoids into the PAG and RVM produces antinociception to thermoceptive stimuli, reversed by selective cannabinoid CB1 receptor antagonists [33,34,42]. Even if intra-PAG cannabinoid-induced antinociception is relatively weaker than that one produced by opioids, opioid treatment results unsatisfactory due to a decrease of spinal opioid receptors whereas cannabinoid effectiveness is associated to an up-regulation of CB1 receptors in certain pathological conditions such as neuropathic pain [43,44]. So far, the mostly used cannabinoid antagonists are the CB1 selective: SR141716A (rimonabant) and AM251 and the CB2 selective: SR144528 and AM630. SR141716A, AM251, SR144528 and AM630 are inverse agonists rather than ‘‘pure’’ or ‘‘silent’’ antagonists [45]. Moreover, AM630 behaves also as a weak partial inverse agonist at CB1 receptor [46] and AM251 might have antagonist/inverse agonist actions at GPR55 [47,48]. Microinjection of a non selective CB1/CB2 receptor agonist, WIN 55,212-2, into the PAG produces an antinociceptive effect which proved to be dependent upon group I metabotropic glutamate subtype 5 (mGlu5) and group II and group III receptor stimulation. Indeed, apart from SR141716A, the effect of WIN55,212-2 was prevented by MPEP (a selective mGlu5 receptor antagonist), ethyl-glutamic acid (a selective group II mGlu receptor antagonist) and MSOP (a selective group III mGlu receptor antagonist) [49]. Pretreatment with a selective antagonist of *N*-methyl-D-aspartate (NMDA) receptors, DL-AP5, blocked the effect of WIN 55,212-2. This evidence suggests that physiological stimulation of mGlu and NMDA receptors is required to produce cannabinoid-induced analgesia in the PAG. In particular, pharmacological blockade of postsynaptic mGlu5 and NMDA receptors might reduce the tonic excitatory control of glutamate on antinociceptive pathways originating from the PAG. The effect of group II and III mGlu receptor antagonists could be mediated by increased GABAergic activity. Thus cannabinoid-induced antinociception, which necessitates of glutamate-induced PAG activation, would be prevented if NMDA, group I and group II and III metabotropic glutamate receptors are blocked. The first clarification of cellular mechanisms of cannabinoid-induced analgesia within the PAG and RVM came from Vaughan *et al.* [50,51]. Tetrahydrocannabinol (THC), WIN 55,212-2 and HU-210 inhibited electrically-evoked and spontaneous miniature GABA_A_ mediated inhibitory postsynaptic currents (IPSCs) in PAG and RVM neurons. The cannabinoid-induced inhibition of synaptic transmission proved to be mediated by cannabinoid CB1 receptors since it was reversed by SR141716A and AM251, while not by SR144528 or the opioid receptor antagonist naloxone. Cannabinoids therefore inhibit GABAergic synaptic transmission on identified PAG and RVM output neurons and in doing so are thought to produce analgesia by reducing GABAergic inhibition of output neurons that form part of a descending analgesic pathway [52]. A functional interaction between PAG cannabinoid and mGlu receptors has subsequently been found in the modulation of pain responses monitored simultaneously to the neuronal activities of RVM nociceptive ON- and antinociceptive OFF- cells [53]. These RVM cell populations are characterized by opposite responses to pain stimuli: a burst of activity immediately prior to withdrawal reflexes (ON-cells) or an inhibition of activity immediately prior to withdrawal reflexes (OFF-cells) in the tail flick test [54]. These cells represent an electrophysiological approach for studying centrally acting analgesics. Intra-PAG microinjection of WIN 55,212-2 prevented formalin-induced changes in RVM cell activities and at higher doses, increased the tail flick latencies. WIN 55,212-2 also reduced the tail flick-related onset of the ON-cell burst as well as the duration of the OFF-cell pause. WIN 55,212-2 reduced and enhanced the ongoing activities of the ON- and OFF-cells, respectively. Such effects were prevented by either SR141716A, or by the selective mGlu5 receptor antagonist, MPEP. Conversely, the selective mGlu1 receptor antagonists, CPCCOEt and (*S*)-(+)-α-amino-4-carboxy-2-methylbenzeneacetic acid (LY367385), were ineffective in preventing WIN 55,212-2-induced effects. This study confirms that physiological stimulation of PAG mGlu5, although not of mGlu1 receptors, would appear to be required for the cannabinoid—induced changes in ongoing and pain-related changes (formalin and tail flick) in RVM ON- and OFF-cell activities [53]. A recent study has demonstrated that spillover of synaptically released glutamate, as a result of stress or pain, stimulates mGlu5 receptor inducing endocannabinoid release, which in turn activates presynaptic cannabinoid CB1 receptor. CB1 receptor activation reduces GABAergic tone thus dis-inhibiting the antinociceptive descending pathway. By this subject the neuronal glutamate transporter limits this heterosynaptic endocannabinoid-mediated cross-talk between glutamatergic and GABAergic synapses within the PAG. Neuronal glutamate transporters is likely to play an important role in regulating the endocannabinoid-mediated analgesic functions of the PAG [55]. A recent study by Drew *et al.* has shown that substance P may facilitate descending analgesia in part by enhancing glutamate-mediated excitation and endocannabinoid-mediated disinhibition of PAG-RVM projection neurons, this latter through inhibition of GABAergic transmission. Consistently with a role for endogenous glutamate in this process, this effect was prevented by mGlu5 receptor antagonist [56].

Several studies have demonstrated that stimulation of group I mGlu receptors promotes the biosynthesis of the endocannabinoid 2-arachidonoylglycerol (2-AG), but not that of anandamide, via Gq/11 protein-activation and PLC-β-mediated cleavage of membrane phosphoinositides, which yields 1,2-diacylglycerol (1,2-DAG), followed by diacylglycerol lipase (DGL)-catalyzed conversion of 1,2-DAG to 2-AG [57,58,59] mechanism. Endocannabinoids may be released in this way from postsynaptic neurons, diffuse across the synaptic cleft and bind to CB1 receptors on presynaptic terminals to regulate calcium and potassium channel activities and ensuing neurotransmitter release [60,61]. This retrograde signaling process seems to be widespread [62] in a variety of regions of the brain, including the striatum [62], hippocampus [63,64,65,66,67,68], midbrain [69], and amygdala [70,71]. On this subject, the reciprocal interactions between cannabinoid and metabotropic glutamate receptors may converge into a unique synaptical mechanism and could have relevant implications such as their simultaneous manipulation to produce analgesia.

## 4. Endocannabinoids within the Antinociceptive Descending Pathway

Electrical stimulation of the dorsal and lateral PAG produces analgesia that is unresponsive to blockade by opioid antagonists [72], is mediated by endocannabinoids, and blocked by CB1 receptor antagonists. Moreover, such electrical stimulation of the dorsal and lateral PAG resulted in anandamide mobilization and CB1 receptor -mediated analgesia [73]. It has been recently shown that 2-AG and anandamide are elevated in dorsal PAG concomitantly with non-opioid stress-induced analgesia (SIA) expression [74]. Moreover, microinjections within the PAG and RVM of inhibitors of fatty acid amide hydrolase (FAAH), the enzyme responsible of anandamide hydrolysis, such as URB597 [74] and arachidonoylserotonin (AA-5-HT) [75] enhanced SIA. Microinjection into the PAG of an inhibitor of the monoacylglycerol lipase, MGL, also induced enhancement of stress-induced antinociception which was CB1 receptor-dependent and associated with elevated levels of 2-AG (but not of anandamide). Apart from CB1 receptors, anandamide also stimulates a transient receptor potential vanilloid type 1 receptor (TRPV1). The effect of FAAH inhibitors may therefore be also due to this “alternative” endocannabinoid receptor involvement. In one of our recent studies, microinjections of URB597 into the ventrolateral PAG, by elevating both anandamide and 2-AG, induced a biphasic effect on thermal nociception and RVM ON and OFF cell activity via preferential CB1 and TRPV1 receptor activation. Throughout a set of pharmacological manipulation it has been determined that TRPV1 stimulation led to antinociception, whilst a low dose of URB597 facilitated nociception via CB1 stimulation (blocked by AM251) [75]. This effect was associated with enhanced or reduced activity of RVM OFF cells, suggesting that these effects occur via stimulation or inhibition of excitatory PAG output neurons, respectively. However, the highest doses of URB597 or WIN 55,212-2, caused only CB1-mediated analgesia associated with disinhibition of RVM OFF and inhibition of ON cells. Within ventrolateral PAG several neurons were found coexpressing TRPV1 and CB1 receptors. The study underlines bidiretional endocannabinoid control within the antinociceptive descending pathway, which through TRPV1 or CB1 receptor stimulation may differentially modulate pain perception [75].

Encannabinoid tone may be modified under certain pathological conditions such as chronic pain. The development of a sensitive method for measuring cannabinoids by atmospheric pressure-chemical ionization mass spectrometry, associated with microdialysis, has made it possible to show that electrical stimulation of the dorsal and lateral PAG produces CB1 receptor-mediated analgesia, accompanied by a marked increase in the release of anandamide. Furthermore, pain triggered by subcutaneous injections of the chemical irritant formalin substantially increased the release of anandamide in the PAG. These findings indicate that the endogenous cannabinoids, such as anandamide, play an important role in a cannabinergic pain-suppression system existing within the dorsal and lateral PAG [73]. Recent studies have shown that levels of anandamide and 2-AG, as well as those of the analgesic anandamide congener, palmitoylethanolamide (PEA), increased in three brain areas of the descending antinociceptive system, i.e. the dorsal raphe (DR), PAG and RVM, as well as in the spinal cord (SC) following chronic constriction injury (CCI) of the sciatic nerve in the rat [76]. Anandamide and 2-AG operating at both spinal and supra-spinal levels are therefore up-regulated during neuropathic pain and it is possible that substances that inhibit both endocannabinoid and PEA inactivation might be useful in the treatment of neuropathic pain. It has also been demonstrated that in neuropathic pain conditions the endocannabinoid and serotonergic systems proved hyperactive in the DR. Repeated stimulation of CB1 receptors with exogenous compounds restores DR serotonergic activity, as well as thermal and mechanical nociceptive thresholds to normal conditions [77]. Normalization of pain-induced over-activation of DR extracellular 5-HT by exogenous cannabinoids opens up the perspective that cannabinoid treatment could also resolve the negative emotional consequences of neuropathic pain, thus representing a possible and very promising advance in the clinical management of this highly debilitating condition. 

## 5. The Development of Novel Analgesic Agents Targeting both Fatty Acid Amide Hydrolase and TRPV1 Receptors

Compounds targeting more than one biological process may be more effective or provide a better safety profile than compounds that are selective on a unique target. Arachidonoylserotonin (AA-5-HT) is a “hybrid” compound with a dual function. It is an FAAH inhibitor [78] which has been shown to increase both peripheral and central AEA levels *in vivo* [79,80] and whose actions proved to be blocked by a CB1 receptor antagonist/inverse agonists [75,81]. AA-5-HT is also a potent (mid-nanomolar) antagonist of TRPV1 receptors expressed in HEK-293 cells [75], and this could contribute to the efficacy of the compound in models of both inflammatory (rat) and neuropathic pain (anti-allodynic effect, rat) [75]. The effects of AA-5-HT were examined after its administration intra-PAG. As expected, AA-5-HT increased endocannabinoid levels in the PAG and induced analgesia. It depressed the RVM ON cell activities as well as those of the OFF cells. The effect of AA-5-HT was mimicked by co-injecting URB597 (a selective FAAH inhibitor) and I-RTX (the selective TRPV1 antagonisint). This drug combination also produced analgesia associated with an inhibition of ongoing ON and OFF cell activities. It has been suggested that the recruitment of “alternative” pathways, such as PAG-locus coeruleus (LC)-spinal cord might be responsible for the AA-5-HT effect [82]. Evidence that intra-PAG AA-5-HT increased LC neuron firing activities and intrathecal phentolamine or ketanserin prevented the analgesic effect of AA-5-HT indeed confirmed this hypothesis. Intra-PAG AA-5-HT also prevented the changes in the firing activity of ON and OFF cells induced by formalin intra-paw, and it inverted the formalin-induced increase in LC adrenergic cell activity. All AA-5-HT effects were antagonized by cannabinoid CB1 and TRPV1 receptor antagonists, thus suggesting that co-localization of these receptors in the PAG could be an appropriate neural substrate for AA-5-HT-induced analgesia [82].

## 6. Conclusions

The ability of cannabinoids to induce antinociception in every animal model has encouraged researchers to gain a greater understanding of this non-opioid analgesic system. Neuroanatomical and site-specific pharmacological studies have revealed the presence of CB1, endocannabinoids and endocannabinoid-degrading enzymes at supraspinal level and in particular within the PAG*-*RVM system, which has been the focus of many pain studies in recent years. Within this circuitry cannabinoids produce analgesia through the dis-inhibition of PAG glutamatergic projecting neurons that results in the stimulation of RVM OFF cells and inhibition of the ON cells. Cannabinoid agonists and FAAH inhibitors of at this level have proved to be analgesics. Moreover, novel compounds with a dual target, such as FAAH inhibition and TRPV1 receptor blockade, may cause analgesic effects that are stronger than they would be if acting on each single target, individually, due to the different respective roles and mechanisms of action in the control of nociception,. With respect to this, hybrid compounds acting on dual targets may prove to be effective where “classic” analgesics have failed. Greater understanding of the cannabinoid mechanisms of action underlying PAG-RVM-induced analgesia could be a step forward in the search for alternative pathological pain treatment.

## References

[1] Iversen L., Chapman V. (2002). Cannabinoids: a real prospect for pain relief?. Curr. Opin. Pharmacol..

[2] Pertwee R.G. (2001). Cannabinoid receptors and pain. Prog. Neurobiol..

[3] Maione S., Starowicz K., Palazzo E., Rossi F., Di Marzo V. (2006). The endocannabinoid and endovanilloid systems and their interactions in neuropathic pain. Drug Dev. Res..

[4] Rinaldi-Carmona M., Barth F., Heaulme M., Shire D., Calandra B., Congy C., Martinez S., Maruani J., Neliat G., Caput D. (1994). SR141716A, a potent and selective antagonist of the brain cannabinoid receptor. FEBS Lett..

[5] Hanus L., Breuer A., Tchilibon S., Shiloah S., Goldenberg D., Horowitz M., Pertwee R.G., Ross R.A., Mechoulam R., Fride E. (1999). HU-308: a specific agonist for CB(2), a peripheral cannabinoid receptor. Proc. Natl. Acad. Sci. USA.

[6] Zimmer A., Zimmer A.M., Hohmann A.G., Herkenham M., Bonner T.I. (1999). Increased mortality, hypoactivity, and hypoalgesia in cannabinoid CB1 receptor knockout mice. Proc. Natl. Acad. Sci. USA.

[7] Ledent C., Valverde O., Cossu C., Petitet F., Aubert L.F., Beslot F., Bohme G.A., Imperato A., Pedrazzini T., Roques B.P. (1999). Unresponsiveness to cannabinoids and reduced addictive effects of opiates in CB1 receptor knockout mice. Science.

[8] Svízenská I., Dubový P., Sulcová A. (2008). Cannabinoid receptors 1 and 2 (CB1 and CB2), their distribution, ligands and functional involvement in nervous system structures--a short review. Pharmacol. Biochem. Behav..

[9] Beltramo M., Bernardini N., Bertorelli R., Campanella M., Nicolussi E., Fredduzzi S., Reggiani A. (2006). CB2 receptor-mediated antihyperalgesia: possible direct involvement of neural mechanisms. Eur. J. Neurosci..

[10] Gong J.P., Onaivi E.S., Ishiguro H., Liu Q.R., Tagliaferro P.A., Brusco A., Uhl G.R. (2006). Cannabinoid CB2 receptors: immunohistochemical localization in rat brain. Brain Res..

[11] van Sickle M.D., Duncan M., Kingsley P.J., Mouihate A., Urbani P., Mackie K., Stella N., Makriyannis A., Piomelli D., Davison J.S. (2005). Identification and functional characterization of brainstem cannabinoid CB2 receptors. Science.

[12] Chin C.L., Tovcimak A.E., Hradil V.P., Seifert T.R., Hollingsworth P.R., Chandran P., Zhu C.Z., Gauvin D., Pai M., Wetter J. (2008). Differential effects of cannabinoid receptor agonists on regional brain activity using pharmacological MRI. Br. J. Pharmacol..

[13] Hohmann A.G., Tsou K., Walker J.M. (1998). Cannabinoid modulation of wide dynamic range neurons in the lumbar dorsal horn of the rat by spinally administered WIN55,212-2. Neurosci. Lett..

[14] Kelly S., Chapman V. (2001). Selective cannabinoid CB1 receptor activation inhibits spinal nociceptive transmission in vivo. J. Neurophysiol..

[15] Kelly S., Chapman V. (2003). Cannabinoid CB(1) receptor inhibition of mechanically evoked responses of spinal neurones in control rats, but not in rats with hindpaw inflammation. Eur. J. Pharmacol..

[16] Lichtman A.H., Martin B.R. (1991). Spinal and Supraspinal components of cannabinoid-induced antinociception. J. Pharmacol. Exp. Ther..

[17] Lichtman A.H., Martin B.R. (1991). Cannabinoid-induced antinociception is mediated by a spinal a2-noradrenergic mechanism. Brain Res..

[18] Richardson J.D., Aanonsen L., Hargreaves K.M. (1998). Antihyperalgesic effects of spinal cannabinoids. Eur. J. Pharmacol..

[19] Agarwal N., Pacher P., Tegeder I., Amaya F., Constantin C.E., Brenner G.J., Rubino T., Michalski C.W., Marsicano G., Monory K. (2007). Cannabinoids mediate analgesia largely via peripheral type 1 cannabinoid receptors in nociceptors. Nat. Neurosci..

[20] Zygmunt P.M., Petersson J., Andersson D.A., Chuang H., Sørgård M., Di Marzo V., Julius D., Högestätt E.D. (1999). Vanilloid receptors on sensory nerves mediate the vasodilator action of anandamide. Nature.

[21] Pacher P., Bátkai S., Kunos G. (2004). Haemodynamic profile and responsiveness to anandamide of TRPV1 receptor knock-out mice. J. Physiol..

[22] Starowicz K., Nigam S., Di Marzo V. (2007). Biochemistry and pharmacology of endovanilloids. Pharmacol. Ther..

[23] Potenzieri C., Brink T.S., Simone D.A. (2009). Excitation of cutaneous C nociceptors by intraplantar administration of anandamide. Brain Res..

[24] Singh Tahim A., Sántha P., Nagy I. (2005). Inflammatory mediators convert anandamide into a potent activator of the vanilloid type 1 transient receptor potential receptor in nociceptive primary sensory neurons. Neuroscience.

[25] Watanabe H., Vriens J., Prenen J., Droogmans G., Voets T., Nilius B. (2003). Anandamide and arachidonic acid use epoxyeicosatrienoic acids to activate TRPV4 channels. Nature.

[26] Vriens J., Owsianik G., Fisslthaler B., Suzuki M., Janssens A., Voets T., Morisseau C., Hammock B.D., Fleming I., Busse R. (2005). Modulation of the Ca2 permeable cation channel TRPV4 by cytochrome P450 epoxygenases in vascular endothelium. Circ. Res..

[27] Grant A.D., Cottrell G.S., Amadesi S., Trevisani M., Nicoletti P., Materazzi S., Altier C., Cenac N., Zamponi G.W., Bautista-Cruz F. (2007). Protease-activated receptor 2 sensitizes the transient receptor potential vanilloid 4 ion channel to cause mechanical hyperalgesia in mice. J. Physiol..

[28] Todaka H., Taniguchi J., Satoh J., Mizuno A., Suzuki M. (2004). Warm temperature-sensitive transient receptor potential vanilloid 4 (TRPV4) plays an essential role in thermal hyperalgesia. J. Biol. Chem..

[29] Akopian A.N., Ruparel N.B., Jeske N.A., Patwardhan A., Hargreaves K.M. (2009). Role of ionotropic cannabinoid receptors in peripheral antinociception and antihyperalgesia. Trends Pharmacol. Sci..

[30] Jordt S.E., Bautista D.M., Chuang H.H., McKemy D.D., Zygmunt P.M., Högestätt E.D., Meng I.D., Julius D. (2004). Mustard oils and cannabinoids excite sensory nerve fibres through the TRP channel ANKTM1. Nature.

[31] Hohmann A.G., Tsou K., Walker J.M. (1999). Cannabinoid suppression of noxious heat-evoked activity in wide dynamic range neurons in the lumbar dorsal horn of the rat. J. Neurophysiol..

[32] Martin W.J., Lai N.K., Patrick S.L., Tsou K., Walker J.M. (1993). Antinociceptive actions of cannabinoids following intraventricular administration in rats. Brain Res..

[33] Lichtman A.H., Cook S.A., Martin B.R. (1996). Investigation of brain sites mediating cannabinoid-induced antinociception in rats: evidence supporting periaqueductal gray involvement. J. Pharmacol. Exp. Ther..

[34] Martin W.J., Coffin P.O., Attias E., Balinsky M., Tsou K., Walker J.M. (1999). Anatomical basis for cannabinoid-induced antinociception as revealed by intracerebral microinjections. Brain Res..

[35] Martin W.J., Patrick S.L., Coffin P.O., Tsou K., Walker J.M. (1995). An examination of the central sites of action of cannabinoid-induced antinociception in the rat. Life Sci..

[36] Martin W.J., Tsou K., Walker J.M. (1998). Cannabinoid receptor-mediated inhibition of the rat tail-fl ick reflex after microinjection into the rostral ventromedial medulla. Neurosci. Lett..

[37] Finn D.P., Jhaveri M.D., Beckett S.R., Roe C.H., Kendall D.A., Marsden C.A., Chapman V. (2003). Effects of direct periaqueductal grey administration of a cannabinoid receptor agonist on nociceptive and aversive responses in rats. Neuropharmacology.

[38] Reynolds D.V. (1969). Surgery in the rat during electrical analgesia induced by focal brain stimulation. Science.

[39] Herkenham M., Lynn A.B., Johnson M.R., Melvin L.S., de Costa B.R., Rice K.C. (1991). Characterization and localization of cannabinoid receptors in rat brain: a quantitative *in vitro* autoradiographic study. J. Neurosci..

[40] Matsuda L.A., Bonner T.I., Lolait S.J. (1993). Localization of cannabinoid receptor mRNA in rat brain. J. Comp. Neurol..

[41] Tsou K., Brown S., Sañudo-Peña M.C., Mackie K., Walker J.M. (1998). Immunohistochemical distribution of cannabinoid CB1 receptors in the rat central nervous system. Neuroscience.

[42] Meng I.D., Manning B.H., Martin W.J., Fields H.L. (1998). An analgesia circuit activated by cannabinoids. Nature.

[43] Siegling A., Hofmann H.A., Denzer D., Mauler F., De Vry J. (2001). Cannabinoid CB(1) receptor upregulation in a rat model of chronic neuropathic pain. Eur. J. Pharmacol..

[44] Zhang J., Hoffert C., Vu H.K., Groblewski T., Ahmad S., O'Donnell D. Induction of CB2 receptor expression in the rat spinal cord of neuropathic but not inflammatory chronic pain models. Eur. J. Neurosci..

[45] Pertwee R.G. (2005). Inverse agonism and neutral antagonism at cannabinoid CB1 receptors. Life Sci..

[46] Ross R.A., Brockie H.C., Stevenson L.A., Murphy V.L., Templeton F., Makriyannis A., Pertwee R.G. (1999). Agonist-inverse agonist characterization at CB1 and CB2 cannabinoid receptors of L759633, L759656, and AM630. Br. J. Pharmacol..

[47] Ryberg E., Larsson N., Sjögren S., Hjorth S., Hermansson N.O., Leonova J., Elebring T., Nilsson K., Drmota T., Greasley P.J. (2007). The orphan receptor GPR55 is a novel cannabinoid receptor. Br. J. Pharmacol..

[48] Mackie K. (2008). Signaling via CNS cannabinoid receptors. Mol. Cell Endocrinol..

[49] Palazzo E., Marabese I., de Novellis V., Oliva P., Rossi F., Berrino L., Rossi F., Maione S. (2001). Metabotropic and NMDA glutamate receptors participate in the cannabinoid-induced antinociception. Neuropharmacology.

[50] Vaughan C.W., McGregor I.S., Christie M.J. (1999). Cannabinoid receptor activation inhibits GABAergic neurotransmission in rostral ventromedial medulla neurons *in vitro*. Br. J. Pharmacol..

[51] Vaughan C.W., Connor M., Bagley E.E., Christie M.J. (2000). Actions of cannabinoids on membrane properties and synaptic transmission in rat periaqueductal gray neurons *in vitro*. Mol. Pharmacol..

[52] Fields H.L., Basbaum A.I., Heinricher M.M., McMahon S.B., Koltzenburg M. (2006). Central Nervous Systems Mechanisms of Pain Modulation. Textbook of Pain.

[53] de Novellis V., Mariani L., Palazzo E., Vita D., Marabese I., Scafuro M., Rossi F., Maione S. (2005). Periaqueductal grey CB1 cannabinoid and metabotropic glutamate subtype 5 receptors modulate changes in rostral ventromedial medulla neuronal activities induced by subcutaneous formalin in the rat. Neuroscience.

[54] Fields H.L., Bry J., Hentall I., Zorman G. (1983). The activity of neurons in rostral medulla of the rat during withdrawal from noxious heat. J. Neurosci..

[55] Drew G.M., Mitchell V.A., Vaughan C.W. (2008). Glutamate spillover modulates GABAergic synaptic transmission in the rat midbrain periaqueductal grey via metabotropic glutamate receptors and endocannabinoid signaling. J. Neurosci..

[56] Drew G.M., Lau B.K., Vaughan C.W. (2009). Substance P drives endocannabinoid-mediated disinhibition in a midbrain descending analgesic pathway. J. Neurosci..

[57] Bisogno T., Howell F., Williams G., Minassi A., Cascio M.G., Ligresti A., Matias I., Schiano-Moriello A., Paul P., Williams E.J. (2003). Cloning of the first sn1-DAG lipases points to the spatial and temporal regulation of endocannabinoid signaling in the brain. J. Cell Biol..

[58] Hashimotodani Y., Ohno-Shosaku T., Tsubokawa H., Ogata H., Emoto K., Maejima T., Araishi K., Shin H.S., Kano M. (2005). Phospholipase Cbeta serves as a coincidence detector through its Ca^2+^ dependency for triggering retrograde endocannabinoid signal. Neuron.

[59] Stella N., Schweitzer P., Piomelli D. (1997). A second endogenous cannabinoid that modulates long-term potentiation. Nature.

[60] Freund T.F., Katona I., Piomelli D. (2003). Role of endogenous cannabinoids in synaptic signaling. Physiol. Rev..

[61] Chevaleyre V., Takahashi K.A., Castillo P.E. (2006). Endocannabinoid-mediated synaptic plasticity in the CNS. Annu. Rev. Neurosci..

[62] Gerdeman G.L., Ronesi J., Lovinger D.M. (2002). Postsynaptic endocannabinoid release is critical to long-term depression in the striatum. Nat. Neurosci..

[63] Varma N., Carlson G.C., Ledent C., Alger B.E. (2001). Metabotropic glutamate receptors drive the endocannabinoid system in hippocampus. J. Neurosci..

[64] Ohno-Shosaku T., Tsubokawa H., Mizushima I., Yoneda N., Zimmer A., Kano M. (2002). Presynaptic cannabinoid sensitivity is a major determinant of depolarization-induced retrograde suppression at hippocampal synapses. J. Neurosci..

[65] Robbe D., Kopf M., Remaury A., Bockaert J., Manzoni O.J. (2002). Endogenous cannabinoids mediate long-term synaptic depression in the nucleus accumbens. Proc. Natl. Acad. Sci. USA.

[66] Brown S.P., Brenowitz S.D., Regehr W.G. (2003). Brief presynaptic bursts evoke synapse-specific retrograde inhibition mediated by endogenous cannabinoids. Nat. Neurosci..

[67] Chevaleyre V., Castillo P.E. (2003). Heterosynaptic LTD of hippocampal GABAergic synapses: a novel role of endocannabinoids in regulating excitability. Neuron.

[68] Rouach N., Nicoll R.A. (2003). Endocannabinoids contribute to short-term but not long-term mGluR-induced depression in the hippocampus. Eur. J. Neurosci..

[69] Marsicano G., Wotjak C.T., Azad S.C., Bisogno T., Rammes G., Cascio M.G., Hermann H., Tang J., Hofmann C., Zieglgänsberger W. (2002). The endogenous cannabinoid system controls extinction of aversive memories. Nature.

[70] Melis M., Pistis M., Perra S., Muntoni A.L., Pillolla G., Gessa G.L. (2004). Endocannabinoids mediate presynaptic inhibition of glutamatergic transmission in rat ventral tegmental area dopamine neurons through activation of CB1 receptors. J. Neurosci..

[71] Azad S.C., Monory K., Marsicano G., Cravatt B.F., Lutz B., Zieglgänsberger W., Rammes G. (2004). Circuitry for associative plasticity in the amygdala involves endocannabinoid signaling. J. Neurosci..

[72] Cannon J.T., Prieto G.J., Lee A., Liebeskind J.C. (1982). Evidence for opioid and non-opioid forms of stimulation-produced analgesia in the rat. Brain Res..

[73] Walker J.M., Huang S.M., Strangman N.M., Tsou K., Sañudo-Peña M.C. (1999). Pain modulation by release of the endogenous cannabinoid anandamide. Proc. Natl. Acad. Sci. USA.

[74] Hohmann A.G., Suplita R.L., Bolton N.M., Neely M.H., Fegley D., Mangieri R., Krey J.F., Walker J.M., Holmes P.V., Crystal J.D. (2005). An endocannabinoid mechanism for stress-induced analgesia. Nature.

[75] Maione S., Bisogno T., de Novellis V., Palazzo E., Cristino L., Valenti M., Petrosino S., Guglielmotti V., Rossi F., Di Marzo V. (2006). Elevation of endocannabinoid levels in the ventrolateral periaqueductal grey through inhibition of fatty acid amide hydrolase affects descending nociceptive pathways via both cannabinoid receptor type 1 and transient receptor potential vanilloid type-1 receptors. J. Pharmacol. Exp. Ther..

[76] Petrosino S., Palazzo E., de Novellis V., Bisogno T., Rossi F., Maione S., Di Marzo V. (2007). Changes in spinal and supraspinal endocannabinoid levels in neuropathic rats. Neuropharmacology.

[77] Palazzo E., de Novellis V., Petrosino S., Marabese I., Vita D., Giordano C., Di Marzo V., Mangoni G.S., Rossi F., Maione S. (2006). Neuropathic pain and the endocannabinoid system in the dorsal raphe: pharmacological treatment and interactions with the serotonergic system. Eur. J. Neurosci..

[78] Bisogno T., Melck D., De Petrocellis L., Bobrov Myu, Gretskaya N.M., Bezuglov V.V., Sitachitta N., Gerwick W.H., Di Marzo V. (1998). Arachidonoylserotonin and other novel inhibitors of fatty acid amide hydrolase. Biochem. Biophys. Res. Commun..

[79] Capasso R., Matias I., Lutz B., Borrelli F., Capasso F., Marsicano G., Mascolo N., Petrosino S., Monory K., Valenti M., Di Marzo V., Izzo A.A. (2005). Fatty acid amide hydrolase controls mouse intestinal motility in vivo. Gastroenterology.

[80] de Lago E., Petrosino S., Valenti M., Morera E., Ortega-Gutierrez S., Fernandez-Ruiz J., Di Marzo V. (2005). Effect of repeated systemic administration of selective inhibitors of endocannabinoid inactivation on rat brain endocannabinoid levels. Biochem. Pharmacol..

[81] Suplita R.L., Farthing J.N., Gutierrez T., Hohmann A.G. (2005). Inhibition of fatty-acid amide hydrolase enhances cannabinoid stress-induced analgesia: sites of action in the dorsolateral periaqueductal gray and rostral ventromedial medulla. Neuropharmacology.

[82] de Novellis V., Palazzo E., Rossi F., De Petrocellis L., Petrosino S., Guida F., Luongo L., Migliozzi A., Cristino L., Marabese I. (2008). The analgesic effect of N-arachidonoyl-serotonin,a FAAH inhibitor and TRPV1 receptor antagonist, associated with changes in rostral ventromedial medulla and locus coeruleus cell activity in rats. Neuropharmacology.

